# Expression of *DnaK* and *HtrA* genes under high temperatures and their impact on thermotolerance of a *Salmonella* serotype isolated from tahini product

**DOI:** 10.1186/s43141-019-0005-4

**Published:** 2019-10-07

**Authors:** Reda M. Gaafar, Marwa M. Hamouda, Khalid A. El-Dougdoug, Sameh Fayez Fouad

**Affiliations:** 1 Botany Department, Faculty of Science, Tanta University, Tanta, 31527 Egypt; 2 Microbiology Department, Faculty of Agriculture, Ain Shams University, PO Box 68, Hadayek Shobra 11241, Cairo, Egypt

**Keywords:** *Salmonella*, Thermotolerance, qRT-PCR, *D* value, *Z* value, Relative gene expression

## Abstract

**Background:**

*Salmonella* is considered to be the second largest source of infection in food-borne diseases. It is also considered one of the most important dangers particularly in the meat and dairy industry. Therefore, the main objective of our study was to determine the relationship between thermotolerance of a *Salmonella* serotype and the expression of *DnaK* and *HtrA* genes.

**Results:**

In this study, expression of the two genes *DnaK* and *HtrA* was compared under four different temperatures 37 °C, 42 °C, 50 °C, and 55 °C in two serotypes of *Salmonella enterica* subsp. *enterica*. One of them was isolated from tahini product and identified as *Salmonella enterica* subsp. *enterica* serovar *choleraesuis*. This identified serotype was found to be more thermotolerant than the second serotype (*Salmonella enterica* subsp. *enterica* serovar *typhimurium* (ATCC 13311)), which was used as reference. This conclusion was based on *D* and *Z* values, which were used to compare thermoresistance ability of the two serotypes under four different temperatures 60 °C, 65 °C, 70 °C, and 75 °C. In addition, the results of qRT-PCR showed that after 43 °C (induction temperature), the relative expression (fold change) of *DnaK* and *HtrA* genes increased up to 5 and 47, respectively, comparing to their expression at 37 °C.

**Conclusions:**

Thermotolerance of the identified *S. choleraesuis* serotype showed significantly high expression levels of *DnaK* and *HtrA* genes.

## Background

Salmonellosis is one of the most well-known food-borne diseases that causes annually over one million infections [1]. Thermal treatment of food is considered as the most effective and cheapest method used to eliminate pathogenic microbes such as *Salmonella*, yet the microbial resistance against heat is a very important factor that limits the thermal treatment of food [2]. *Salmonella* is relatively heat resistant as a member of *Enterobacteriaceae* comparing to *E. coli*, where the *D* values of *Salmonella* spp. and *E. coli* in fried poultry meat at 70 °C are 13.2 and 2.5 s, respectively [3]. In addition, the *D* value of *Salmonella* spp. at 70 °C was found to be 24 s at water activity of 0.72 in poultry meat [4] and this is a high thermotolerance degree for a member of *Enterobacteriaceae*.

It is well known that the causes of thermal resistance in bacteria vary greatly from surrounding environmental to genetic causes [5, 6]. In addition, heat shock proteins (HSPs) are noted to play a pivotal role in the survival of bacteria from damage caused by exposure to heat. Moreover, these HSPs are essential to maintain the life of bacteria during exposure to various kinds of stress [7]. A study conducted by Sirsat et al. [8] indicated a correlation between relatively high expression rates of some heat shock genes and thermal tolerance in *Salmonella*. Furthermore, it is well known that *Salmonella* gains more thermoresistance if it is subjected to sublethal temperature and even becomes more severe in virulence [8]. However, less is known about the variability in heat tolerance on the serotype level. It seems that some serotypes are more heat resistant than others, where this variation was due to difference in *D* and *Z* values as observed in *S. senftenberg* and *S. typhimurium* serotypes [9].

One of the heat shock genes is *HtrA* gene. It codes for a degradation protein (DegP), which acts as a molecular chaperone and as a periplasmic endopeptidase enzyme that maintains the periplasm. The mutants of this gene were found to be not able to grow at temperature above 42 °C [10], where *HtrA* mutant *Salmonella* suffers highly attenuated survival during the infection [11]. Another heat shock gene is *DnaK* gene. It codes for HSP70 protein, which is considered as the most important heat shock protein. It is a molecular chaperone, which makes the refolding of the misfolded proteins and disperses the aggregated protein molecules in stress conditions [12]. It was reported that the accumulation of heat shock proteins is directly proportional to the elevation of the temperature. However, major heat shock proteins, including HSP70, were not thought to have direct relationship to the thermotolerance because one protein of molecular weight 34 kDa disappeared rapidly following a temperature downshift (48 °C to 37 °C) proving that this protein is related to thermotolerance and HSPs are not [13]. However, several recent studies found that HSP100 (ClpB) has a crucial role in thermotolerance mechanism [14, 15] by performing a complex with HSP70 heat shock protein [16, 17].

The main objective of this study was to compare the expression of two heat shock genes *DnaK* and *HtrA* under four different temperatures 37 °C, 42 °C, 50 °C, and 55 °C in two serotypes *S. choleraesuis* and *S. typhimurium*. Moreover, thermotolerance of the two *Salmonella* serotypes was evaluated and compared.

## Methods

### Isolation of *Salmonella*

Thirty-two samples of tahini product from a production line, which was known to be suffering from contamination with *Salmonella*, were examined for presence of *Salmonella* spp. Examination of samples was done following the ISO standard 6579:2002 [18] (horizontal method for the detection of *Salmonella* spp.). The three main steps of isolation of *Salmonella*, pre-enrichment in buffered peptone medium, selective enrichment in Muller-Kauffmann tetrathionate (MKTT), and Rappaport-Vassiliadis (RV) enrichment broths and streaking on xylose lysine desoxycholate agar (XLD) and Hektone enteric agar, were done followed by biochemical and serological confirmations. All the media used for *Salmonella* isolation were purchased from Oxoid-Thermo Fisher Scientific (UK).

### Molecular identification using 16s RNA

16s RNA gene analysis was performed in Clinilab Laboratories, Al-Maadi, Cairo, Egypt. Bacterial DNA was extracted using a bacterial DNA extraction kit (Qiagen, USA) according to Green and Sambrook [19]. The extracted DNA was treated with RNase A solution (10 ng/μl) to remove any contamination with RNA. Then, PCR was performed using the universal (16s RNA forward and reverse) primers (Table 1), which were designed to amplify 1500 bp fragment of the 16s rRNA gene region. The PCR was carried out for 30 cycles at 94 °C for 1 min, 55 °C for 1 min, and 72 °C for 2 min to each cycle. The PCR amplicon was sequenced using the same amplification primers. Automated DNA sequencing based on enzymatic chain terminator technique [20] was performed using 3130X DNA Sequencer (Genetic Analyzer, Applied Biosystems, Hitachi, Japan). The consensus sequence of forward and reverse reads was then compared with sequences in NCBI GenBank using similarity analysis BLASTN tool. The multiple sequence alignment (MSA) and molecular phylogenetic analyses were performed using BioEdit software [21].

**Table 1:** Sequences of primers used in this study for 16s bacterial identification and for heat shock gene (*DnaK* and *HtrA*) expression analysis

Primer name	Sequence (5′–3′)
16s RNA forward	5′-AACTGGAAGGTGGGGAT-3′
16s RNA reverse	5′-AGGAGGTCCAACCGCA-3′
16s RNA-qRT-PCR-F	5′-TCCTACGGGAGGCAGCAGT-3′
16s RNA-qRT-PCR-R	5′-GGACTACCAGGGTATCTAATCCTGTT-3′
*DnaK-*StF (*S. typhimurium*)	5′-CGCTTCCAGGACGAAGAAGT-3′
*DnaK*-StR (*S. typhimurium*)	5′-CGAGG TCGTAAACCGCGATA-3′
*DnaK-*ScF (*S. choleraesuis*)	5′-CGCTTCCAGGACGAAGAAGT-3′
*DnaK*-ScR (*S. choleraesuis*)	5′-CGAGG TCGTAAACCGCGATA-3′
*HtrA-*StF (*S. typhimurium*)	5′-CGACGAACAACTCTGGCTCA-3′
*HtrA-*StR (*S. typhimurium*)	5′-TTCAAG GGTGTCGAGATGGC-3′
*HtrA-*ScF (*S. choleraesuis*)	5′-GAGTGCACTGGCTCTGAGTT-3′
*HtrA-*ScR (*S. choleraesuis*)	5′-TTCACC GTGGTGCTACCTTC-3′

### Serotype determination

The serotype of the isolated *Salmonella* was determined according to Kauffman-White -Le Minor scheme that was updated in Grimont and Weill [22]. A group of antisera containing omnivalent, polyvalent, and monovalent antisera (Sifin, Berlin, Germany) was used to determine the serotype of the isolated bacteria in three separated tests (three different single colonies). A single colony in each test was first mixed with 1 ml of sterilized 1% NaCl saline solution. Then, one drop of antisera was added and the agglutinations were observed and recorded.

### Assessment of bacterial thermotolerance

In order to determine the thermotolerance of the isolated bacteria, *D* and *Z* values were measured in aqueous solution taking *Salmonella typhimurium* ATCC 13311, taxid: 90371 as a reference [23]. Both bacteria were grown in a nutrient medium: brain heart infusion broth (Oxoid) at 37 °C for 24 h. Then, the bacterial broth was distributed to new fresh brain heart infusion broth media and grown under different temperatures (60 °C, 65 °C, 70 °C, and 75 °C). The count of survival bacteria was done at three time points (1, 2, and 3 min) for each temperature using a differentiative selective medium (brilliant green bile agar) to determine *D* values of bacteria at these temperatures. Using *D* values, a graph was constructed for *Z* value determination in which *X*-axis represented the logarithm of survival bacterial counts and *Y*-axis showed the temperatures. The *Z* value was obtained by inverting the curve slope. The results of both serotypes were compared using Tukey’s multiple comparisons test of GraphPad Prism6 software, where alpha = 0.05 was selected.

### *DnaK* and *HtrA* expression analysis

#### RNA extraction

To extract the total RNA, bacterial cells were first exposed to 37 °C, 42 °C, 47 °C, 50 °C, and 55 °C for 18 h, and then, total RNA was extracted using RNeasy Mini Kit, cat. no.: 74104 (Qiagen, Valencia, CA, USA). Afterwards, RNA extract was treated with RNase-free DNase (Qiagen-DNA wipe out) to remove residual genomic DNA. Subsequently, the verification of RNA extraction was performed using 1.5% agarose gel electrophoresis. The purified RNA was reverse transcribed into cDNA using reverse transcription Qiagen kit (QuantiTect® Reverse Transcription, cat. no.: 205311). Finally, the obtained cDNA was verified using 1.5% agarose gel electrophoresis.

#### Quantitative real-time PCR

To measure the relative expression of the target genes (*DnaK* and *HtrA*), quantitative real-time PCR (qRT-PCR) assay was performed on cDNA using the gene-specific primers (Table 1) and a ready-to-use qRT-PCR kit (QuantiTect SYBR Green PCR). The transcripts of these two genes were analyzed on Rotor-Gene Q48 Thermocycler. The 16s RNA gene (housekeeping gene) expression was used as a reference gene and analyzed in parallel using specific primers (16s RNA-qRT-PCR-F and R) of 16s RNA gene of Gram-negative bacteria (Table 1). Calculation of the relative gene expression was done according to 2^-ΔΔCt^ method for normalizing the cycle threshold values [8, 24]. The gene expression of *DnaK* and *HtrA* in both serotypes was compared using Tukey’s multiple comparisons test of GraphPad Prism6 software, where alpha = 0.05 was selected.

## Results and discussion

### Bacterial isolate identification

Four samples (*Salmonella*-positive samples) of the total 32 examined tahini samples were found to be infected with *Salmonella*. These isolated bacteria gave the specific characteristics of *Salmonella*: dark colonies with black center on XLD, black colonies on Hektoen enteric agar, negative urease reaction on urea agar, sulfite reduction on triple sugar iron agar (TSI) and lysine iron agar (LI) with gas formation in TSI, negative β-galactosidase, negative indole, and negative Voges-Proskauer test. The confirmatory tests using poly O-Vi (somatic and capsular) antisera and poly H (flagellar) antisera were performed, and the results showed that the isolated bacteria are *Salmonella*.

For molecular identification of the *Salmonella* isolates, the 16s RNA gene analysis was performed. The PCR fragment of 475 bp was amplified and sequenced. Then, sequence BLASTN analysis was performed on the GenBank (NCBI) databases. A 99% sequence similarity was found to be with *Salmonella enterica* subsp. *enterica,* which indicates that the isolated bacteria are *Salmonella enterica* subsp. *enterica* serotype *choleraesuis*. The sequence of 16s gene of the isolated bacteria was deposited in NCBI GenBank and was given the following accession no. MK041288.1.

Moreover, serological analysis was performed to determine the specific serotype as the 16s RNA analysis is not a precise method at the levels lower than species in bacteria. Therefore, full *Salmonella* serological identification was performed on three isolates from the four positive samples. The results of the full serological tests showed that the antigenic formula of the three bacterial isolates is somatic antigen 6, 7; flagellar antigen phase 1: c; and flagellar antigen phase 2: 1, 5. By referring to the Kaufmann-White-Le Minor scheme [22], the bacterial serotype was confirmed as *Salmonella enterica* subsp. *enterica* serovar *choleraesuis.*

For identification of *Salmonella* serotypes, till now, serotyping using antisera and pulsed-field gel electrophoresis (PFGE) is the only accredited traditional methods. However, serotyping using antisera has an advantage of being cheaper than any other DNA dependent methods. Even the PFGE method is still expensive and takes much time for comparing bacterial serotypes [25]. In addition, PFGE does not show a unified profile for different strains of the same serotype [26]. On the other hand, the rates of concordance at the species level by using partial sequencing 16s rRNA gene and sequence alignment technique were found to be 80% [27]. Therefore, it seems that using 16s rRNA analysis is not trusted at taxonomic levels lower than species. So, in this study, we considered the partial sequencing of 16s rRNA gene as a primary identification method and the results were confirmed using the serotyping procedure. Some new serotype identification techniques are depending on 16s rRNA like 16s rRNA PCR-high-resolution melt analysis assay (HRMA), which is quite accurate [28] however it still needs more data to cover all serotypes of *Salmonella* [29]. Therefore, about 2600 serotypes are reported because of limited resolution and lower sensitivity of 16s rRNA gene analysis compared to metagenomic sequencing method [30, 31].

### Thermotolerance analysis

In order to evaluate the thermotolerance of the isolated bacterium (serotype), *S. choleraesuis* in parallel with *Salmonella typhimurium* ATCC 13311 (reference bacterium), *D* value and *Z* value determination of both serotypes in aqueous nutrient solutions at water activity of 0.98 was performed under different temperatures 60 °C, 65 °C, 70 °C, and 75 °C. The results showed that the *D* values of *S. choleraesuis* at the previously mentioned temperatures are 45.6, 33.6, 21.6, and 9.6 s, respectively, while the corresponding *D* values for *S. typhimurium* were 43.2, 35.4, 16.2, and 3.6 s, respectively. The *Z* values were found to be 4.9 and 4.5, respectively. Therefore, based on our results, thermotolerance in the isolated strain of *S. choleraesuis* is relatively more than in *S. typhimurium* (ATCC 13311). It looks like that there is a wide range of thermotolerance variability of *Salmonella* among different serotypes and even among different isolates of the same serotype. It was reported that the *D* values of *Salmonella agona* at the temperatures 60 °C, 65 °C, and 70 °C in buffered peptone solution were 148, 19.8, and 7.8 s, respectively. These values are considered very high *D* values for this serotype of *Salmonella enterica*, while the *D* values of *Salmonella typhimurium* at the same temperatures were 13.2, 6, and 1.2 s. It has been shown that the *Z* value of *Salmonella* spp. ranged between 3.9 and 7.4 °C [32] which is in accordance with the *Z* value obtained in our study. However, their *D* values are still higher and lower compared to our results.

It has been reported that the *D* values at 60 °C and 65 °C of *Salmonella* spp. in beef meat were 8.6 min and 1.5 min, respectively [4], while according to [34–36], the *D* values of *Salmonella* spp. at the same temperatures and in the same product were 5.3 and 0.53 min, respectively [33], indicating variability in the *D* values of *Salmonella* spp. The main cause of the high values in the previously mentioned study could be due to lower water activity. In addition, Liu et al. [4] showed that 70 °C is a critical temperature for *Salmonella* death, which is coherent with the results of the present study. Moreover, high thermotolerance of *Salmonella* in the aqueous solutions with high water activity was reported, where the heat treatment up to 85 °C for 1 min did not eliminate the naturally occurring contaminant from alfalfa seeds [34]. This finding is in parallel with our results indicating that *Salmonella* can endure high temperatures in aqueous solutions.

**Fig. 1:**
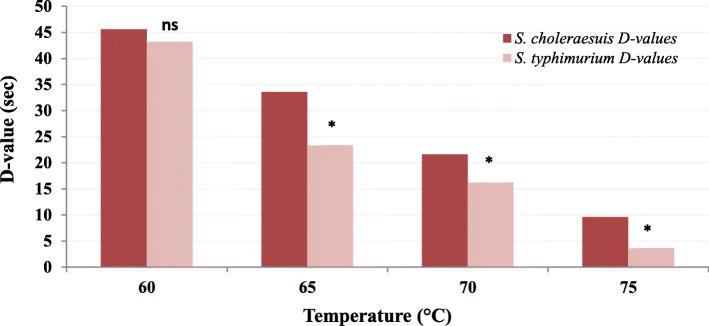
Histogram comparing the *D* values of *S. typhimurium* and *S. choleraesuis* grown under different temperatures. *Significant difference between both bacteria at temperature. ns, no significant difference

**Fig. 2:**
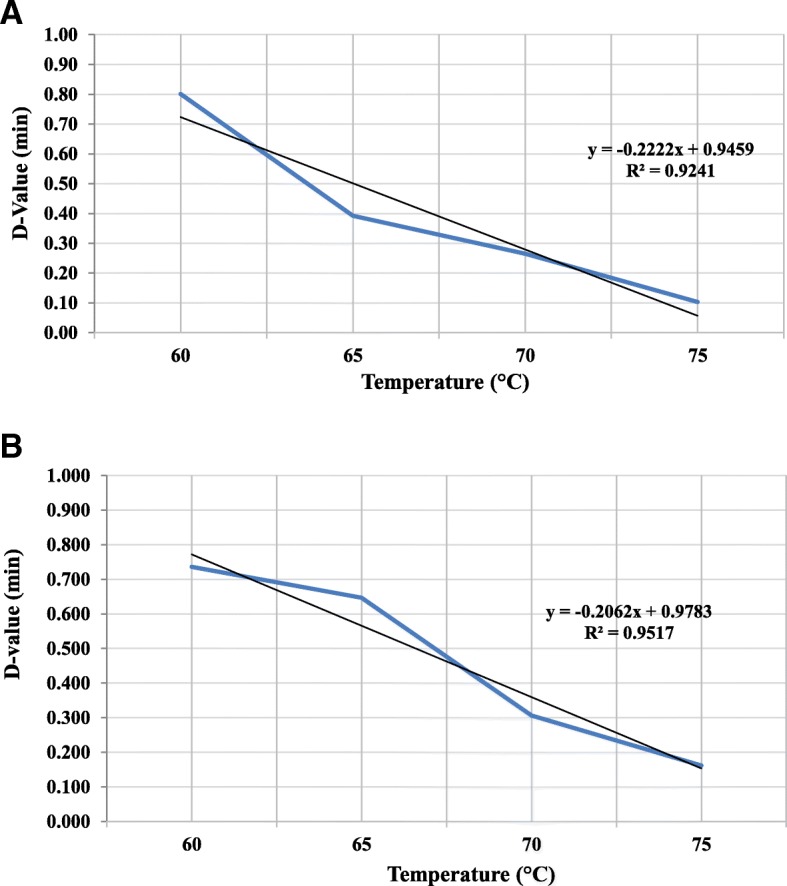
*Z* value determination curves of *S. typhimurium* (**a**) and *S. choleraesuis* (**b**) grown under different temperatures

In our study, statistical analysis showed a significant difference between the *D* values of both bacteria *S. choleraesuis* and *S. typhimurium* under three temperatures 65 °C, 70 °C, and 75 °C (Fig. 1). Also, the thermotolerance superiority of *S. choleraesuis* on *S. typhimurium* was found. In addition, there was a significant difference between the obtained *Z* values of both serotypes (were determined depending on the obtained *D* values) 4.9 and 4.5 degree, respectively (Fig. 2). Growing both serotypes at the same conditions indicated that the difference in *D* and *Z* values is due to genetic factors and it is neither temporarily acquired nor due to the environmental conditions. These obtained results are consistent with the findings of Alvarez et al. [9], Dodier [35], and de Melo et al. [36], where it was reported that there is a difference in thermotolerance among *S. senftenberg*, *S. typhimurium*, and *S. enteritidis* serotypes.

### Relative gene expression analysis

**Fig. 3:**
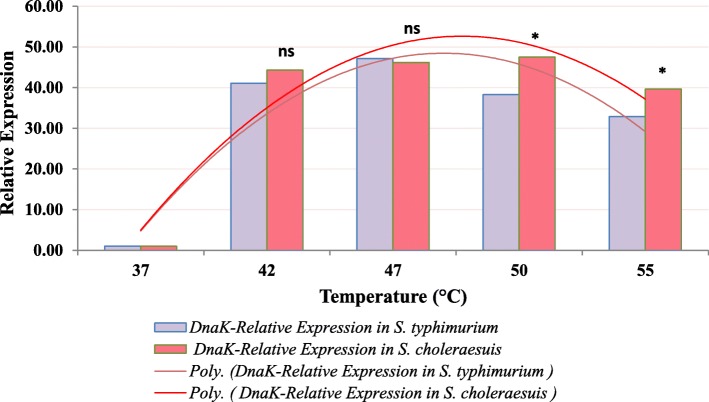
Relative expression of *DnaK* gene in *S. typhimurium* and *S. choleraesuis* grown under different temperatures. *Significant difference between both bacteria at temperature. ns, no significant difference; Poly., polynomial curve

**Fig. 4:**
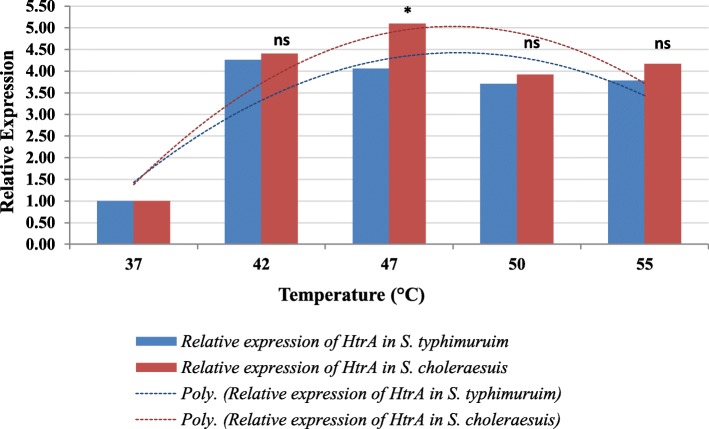
Relative expression of *HtrA* gene in *S. typhimurium* and *S. choleraesuis* grown under different temperatures. *Significant difference between both bacteria at temperature. ns, no significant difference; Poly., polynomial curve

The expression of *DnaK* and *HtrA* genes in both serotypes was investigated to find out if there is a correlation between the expression of these genes and thermotolerance. Relative expression analysis using qRT-PCR was performed on the bacteria grown under different temperatures (37 °C, 42 °C, 47 °C, 50 °C, and 55 °C), while the bacteria grown at 37 °C were used as control. In our study, the relative gene expression of *DnaK* in *S. choleraesuis* at the different temperatures 37 °C (control), 42 °C, 47 °C, 50 °C, and 55 °C were 1.00, 44.32, 46.21, 47.50, and 39.67, respectively (Fig. 3). The highest relative gene expression of *DnaK* was at 50 °C, and then, its expression started to decline but not sharply. By contrast, the *DnaK* gene expression at the different temperatures in *S. typhimurium* were 1.0, 41.07, 47.18, 38.32, and 32.90, respectively. It is obvious that the highest *DnaK* expression in *S. typhimurium* was at 47 °C, and these results are in the same ranges of previous measurements [8]. On the other hand, the *HtrA* relative expression was found to be higher in *S. choleraesuis* than in *S. typhimurium* (Fig. 4). The relative *HtrA* expression was 1.00, 4.41, 5.10, 3.92, and 4.17, respectively, in *S. choleraesuis*, while it was 1.00, 4.26, 4.06, 3.71, and 3.78, respectively, in *S. typhimurium*; this range of gene expression levels is matching with the results of Baron et al. [37], where they reported that the expression fold of *HtrA* after induction increased up to 4.83–7.79 times. The highest *HtrA* expression was at 47 °C in *S. choleraesuis*, while it was at 42 °C in *S. typhimurium*. Therefore, it is clear that the peak of relative gene expression of both heat shock genes (*DnaK* and *HtrA*) comes more lately in *S. choleraesuis* serotype.

Our results of qRT-PCR analysis showed that the relative expression of two studied heat shock genes *DnaK* and *HtrA* increased with increasing temperature. These results are in accordance with the findings of Sirsat et al. [8], where they found that the expression of *HtrA* and *DnaK* were upregulated up to 4.11- and 44-fold, respectively, at temperatures over 40 °C. In addition, in this study, the gene expression of both genes *DnaK* and *HtrA* in both serotypes increased dramatically with increasing temperature. Moreover, when the temperature is shifted from 37 to 42 °C, there was a sudden increase in gene expression indicating a threshold in this range of temperature, which is also coherent with the previously mentioned study [8]. Also, a significant difference in the relative expression of *HtrA* gene between *S. typhimurium* and *S. enteritidis* was reported, which was accompanied with relatively higher thermotolerance of *S. typhimurium* that has the higher gene expression [38]. This result is also in accordance with our results.

Comparing the results of relative gene expression of *DnaK* and *HtrA* in both serotypes using Tukey’s multiple comparisons test indicated that the trend of relative expression of both genes is higher in *S. choleraesuis* than in *S. typhimurium*. Moreover, the difference of relative expression of both genes is significant and relative gene expression has a correlation with the bacterial thermotolerance. This result also meets the results of Yadav et al. [38] in their study on thermotolerance difference between *S. typhimurium* and *S. enteritidis*.

## Conclusions

In this study, the tahini bacterial isolate was biochemically, molecularly, and immunologically identified as *S. choleraesuis* serotype. This identified serotype was found to be more thermotolerant than *S. typhimurium* ATCC 13311 (reference serotype) in aqueous solution with water activity of 0.98, which was due to genetic basis although both serotypes belong to the same genus, species, and subspecies. This difference in thermotolerance was accompanied with higher relative expression of two heat shock genes *DnaK* and *HtrA*, which is probably the reason of relatively high thermotolerance of this bacterial isolate (serotype).

## Data Availability

The datasets used and/or analyzed during the current study are available from the corresponding author on reasonable request.
